# Anthropometric indices among schoolchildren from a municipality in Southern Brazil: a descriptive analysis using the LMS method☆

**DOI:** 10.1016/j.rpped.2014.04.002

**Published:** 2014-12

**Authors:** Valter Cordeiro Barbosa, Adair da Silva Lopes, Ricardo Rosa Fagundes, Wagner de Campos

**Affiliations:** a Universidade Federal de Santa Catarina (UFSC), Florianópolis, SC, Brazil; b Universidade Federal do Paraná (UFPR), Curitiba, PR, Brazil

**Keywords:** Child development, Anthropometry, nutritional status, Cross-sectional study, Children

## Abstract

**OBJECTIVE:**

To describe the percentile values for body mass index (BMI), waist circumference (WC) and waist-to-height (WHtR) of children from Colombo, Brazil, and compare them with data of children from other countries.

**METHODS:**

This was a cross-sectional study with a random sample of 2,035 children aged 6-11 years. Age- and sex-specific smoothed percentiles curves for BMI, WC and WHtR were created using the LMS method. Values of 10^th^, 50^th^ and 90^th^ percentiles from Brazilian children were compared with data from other countries.

**RESULTS:**

There was a trend of increasing BMI and WC with age in both sexes. WHtR remained constant with advancing age in boys and girls. Comparison of the growth pattern among countries showed clear differences. Southern Brazil boys and girls had elevated 90^th^ percentile values for BMI, which was similar to German children and higher than the North American and World Health Organization percentile values. However, children from this study had intermediate values for WC and WHtR in comparison to children from other countries.

**CONCLUSIONS:**

Elevated BMI values were observed among southern Brazilian children, but WC and WHtR percentile values were lower in southern Brazilian children than in children from other countries. Interventions at different levels should be made to avoid a probable increase of nutritional disorders (especially general obesity) in the next years.

## Introduction

Body growth standard indicates the "acceptable" and "expected" body development during childhood, which all children should achieve.1 However, the occurrence of nutritional disorders related to child's growth, such as general or centralized (abdominal) obesity reflects the interaction between unfavorable environmental factors and/or genetic predisposition.^2^ As the general and abdominal obesity are increasingly present in children^3^ and they increase the chances of early^4^^,^ 5 and future^6^ metabolic complications, monitoring of physical growth is crucial in promoting children's health.

The combination of different anthropometric measurements (e.g., weight, height or waist circumference [WC]) or indicators (e.g., body mass index [BMI] and waist-to-height ratio [WHtR]) has been frequently used in studies on child growth and health.^6^^-^ 8 Of these, BMI is the most frequently anthropometric indicator used to identify physical growth pattern and nutritional status in clinical and epidemiological practice because it is a simple and low-cost indicator, as well as it is a strong predictor of child's health.^7^^,^ 8 WC represents the accumulation of abdominal and visceral fat and predicts cardiovascular risk factors as well as or even better than BMI.^4^^,^ 6 Other studies have highlighted that WHtR is strongly associated with cardiovascular risk factors at early ages.^5^^,^ 9 Thus, the use of these different anthropometric methods allows a better estimate of child growth pattern and nutritional status during childhood.

Reference percentile curves were developed to show the growth pattern and the nutritional status of pediatric populations of different countries.^10^^-^ 20 These curves apply the LMS method, a statistical procedure for more robust estimation of percentiles values, especially if the physical growth variables do not have a symmetrical distribution in the population.^21^^,^ 22 Some studies also used the LMS method to compare body mass and height percentiles from Brazilian children with those of other countries.^23^^,^ 24 However, a comparison of BMI, WC and WHtR percentiles values between Brazilian children and children from other countries is unknown. A study with these different anthropometric indicators may represent a better estimate of physical growth pattern among Brazilian children. Also, it is necessary to test whether there is anthropometric difference between children from Brazil and from these countries in order to identify growth pattern distinctions and test the necessity of the anthropometric percentile curves for Brazilian children.^18^ Finally, this comparison may identify if a childhood population has physical growth trends (for environmental and genetic conditions) that favor nutritional disorders such as general and abdominal obesity.^2^ These issues are important for the development of public policies aimed at reducing nutritional disorders in Brazilian children.

Therefore, the aim of this study was to determine the physical growth pattern (BMI, WC and WHtR) among schoolchildren from Colombo, Parana, southern Brazil, and to compare it with the physical growth of children from other countries.

## Method

This was a cross-sectional study conducted in the city of Colombo, state of Parana, southern Brazil. This municipality is located in the northern metropolitan region of Curitiba (the State Capital) and it's Human Development Index (IDH 2000) is 0.764, which is the 107^th^ among 399 municipalities in the State of Parana. Colombo had 27,000 children enrolled in regular classes from 1^st^ to 5^th^ grades in public and private schools. This was the study population.

The following statistical parameters were considered to estimate the sample size: (1) confidence level of 95%, (2) sampling error of 3%, (3) prevalence of obesity (at least one obesity indicator) of 50%, which considers the maximum variance and overestimates the sample size and (4) the design effect of 1.4.^25^ Thus, the minimum sample size of the study was estimated at 1,978 children. A margin of 20% was added for possible losses and refusals during data collection. Therefore, the estimated sample included 2,400 children.

The schools were grouped into three strata: public schools in the urban area, public schools in the rural, and private schools. First, a random selection of schools was performed in each of the three strata. The number of schools by stratum was calculated considering the proportionality of children in each stratum. All selected schools were invited and accepted to participate of this study. Secondly, all children involved in 1^st^ to 5^th^ grades classes of elementary school were invited to participate in the study. We visited 14 schools, which included a total of 138 classes, and 2,750 children who were invited to participate in this study.

We performed the anthropometric measurements (weight, height and WC) from March to September 2012. Each child was assessed individually in order to minimize constraints. All measurements were performed by a single experienced evaluator aiming to exclude inter-measurer errors. Two Physical Education teachers performed the annotation of anthropometric data. 

The materials used in this study included: tape-measure (Easyread Cateb^(r)^, São Paulo, Brazil) (0.1cm wide) fixed on a wall that had no footer to determine the height; digital scale (Wiso^(r)^, Santa Catarina, Brazil) (resolution of 100g and capacity of 150 kg) to measure the body weight; and metal tape-measure (Cescorf^(r)^, Rio Grande do Sul, Brazil) (resolution of 0.1 cm) to assess WC. Height and body mass were measured according to the standard protocol.^26^ For height and body weight measurements, the child was evaluated in the standing position, without shoes and wearing the Physical Education uniform. Measurements of body weight and height were used to calculate BMI (kg/m^2^). BMI was classified by sex and age according to the values proposed by the World Health Organization (WHO).^15^

WC was measured in the standing position at the midpoint between the lower costal border and the iliac crest.^6^ Two measurements were made in each child and the average of them was calculated (intraclass correlation coefficient =0,99). Waist-to-height ratio (WHtR = WC/height) was the calculated.^6^

Age was calculated considering the difference between the birthday and the collection date. The following criterion was used to determine the interval between ages: 6.0-6.9=6; 7.0-7.9=7 years; 8.0-8.9=8; 9.0-9.9=9 years, 10.0-10.9=10 years, and 11.0-11.9=11 years. Gender, type of school, shift, grade and location of the school were determined according to the information obtained from each school board.

Descriptive statistics were based on mean and standard deviation for continuous variables, and absolute and relative frequencies for categorical variables. Age- and sex-specific percentile values curves were constructed ​​for anthropometric variables (BMI, WC and WHtR) using the LMS method.^21^^,^ 22 The LMS method assumes that for positive and ​​independent data, the Box-Cox transformation for each age may be employed to normalize the distribution of values ​​of each anthropometric variable. All age- and sex-specific percentile values ​​for BMI, WC and WHtR (3^rd^, 10^th^, 25^th^, 50^th^, 75^th^, 90^th^ and 97^th)^ were smoothed using the LMSChartmaker Light program version 2.3 (The Institute of Child Health, London: www.healthforallchildren.co.uk). 

Graphs that included the 10^th^, 50^th^ and 90^th^ percentile values of the children from this study and from other studies were constructed. BMI percentile values were compared with data from WHO,^15^ US Center for Disease Control and Prevention,^16 ^Pakistan,^20^ India^13^ and Germany.^17^ WC percentile values were compared with data from Germany,^18^ Pakistan,^14^ Mexico,^11^ Hong Kong^19^ and India.^12^ Finally, WHtR percentile values were compared with those from India,^12^ Hong Kong,^19^ Pakistan^14^ and Norway.^10^ These studies were chosen considering: (1) children with similar age range of this study; (2) same statistical procedures for construction of the percentile curves (LMS method); (3) presence of 10^th^, 50^th^, and 90^th^ percentile values; and (4) same protocols for anthropometric measurements. 

The Ethics Committee on Research of the Federal University of Parana approved the study (CAAE: 5371.0.000.091-10). Each school gave a formal permission to collect the data. Each child's parent/guardian provided written informed consent for participation in the study.

## Results

Among the 2,750 children invited to participate in the study, 26% did not return the consent form signed by parents/guardians or refused to participate. These children were not evaluated. Additionally, one child with 15 years old was excluded. There were no losses due to incomplete data filling. Thus, the final sample was composed of 2,035 children (1,016 boys and 1,019 girls) who had a mean age of 8.9±1.4 years old. The majority of the sample included students who studied in the morning period (55.4%), from public schools (63.3%), and from schools located in the urban area. According to the WHO criteria, 0.8% of children were underweight, 19.4% were overweight and 6.0% were obese.

Considering the age- and sex-specific smoothed percentiles values, BMIincreased with age both in boys and girls. BMI tended to be similar in boys and girls up to 10 years. At age 11, girls had higher BMI values than boys. WC had a slightly increase up to 8 years of age. After this age, there was a faster increase of WC in both boys and girls. WC was similar in boys and girls up to 8 years of age. Girls had a higher WC than boys at posterior ages. WHtR ​​remained stable up to 9 years of age. Minimal reductions in the WHtR values were observed at posterior ages. This trend was similar in boys and girls (Table 1).

**Table 1:**
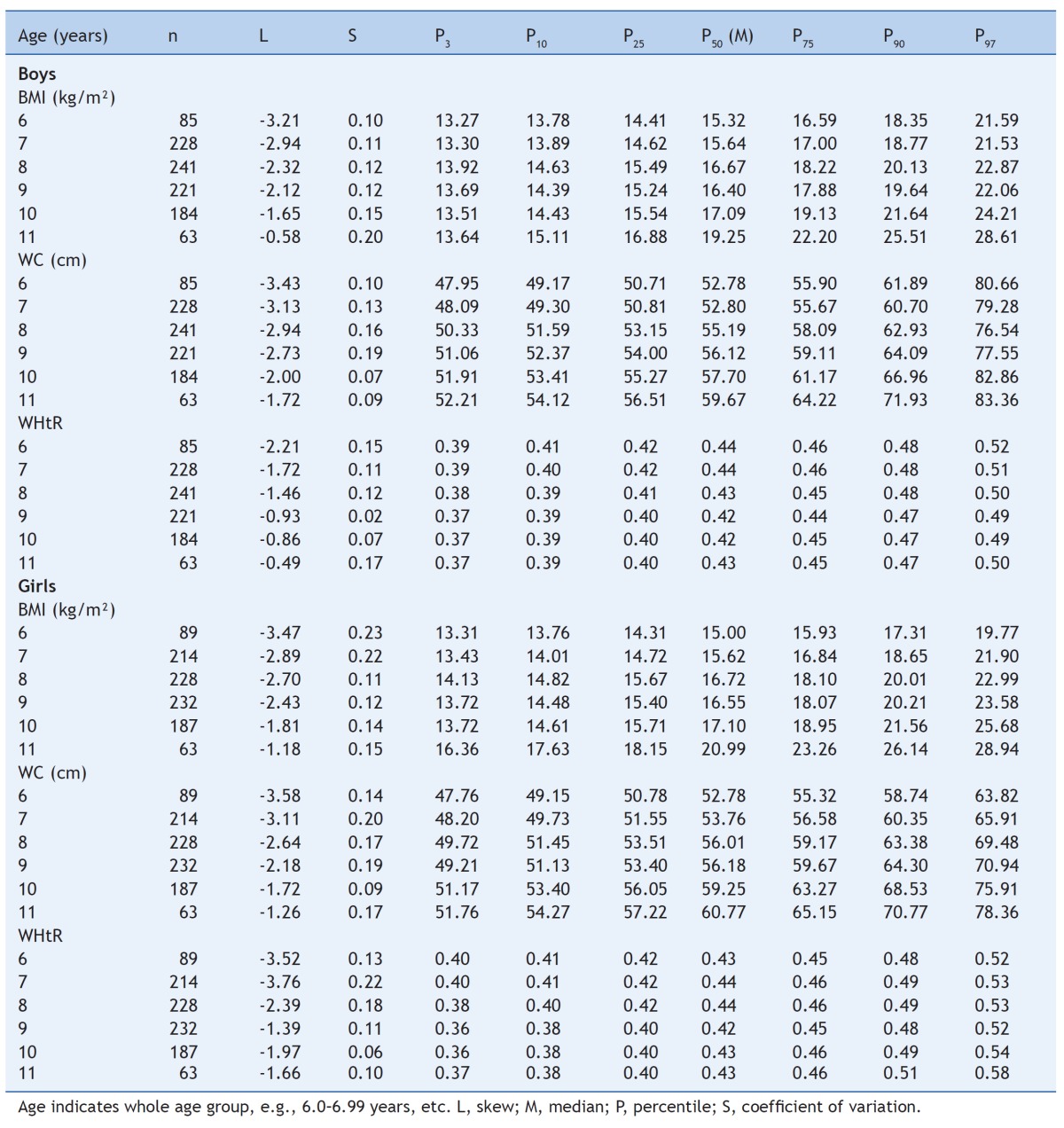
Smoothed age- and sex-specific percentile values for body mass index, waist circumference and waist-height ratio.

**Figure 1:**
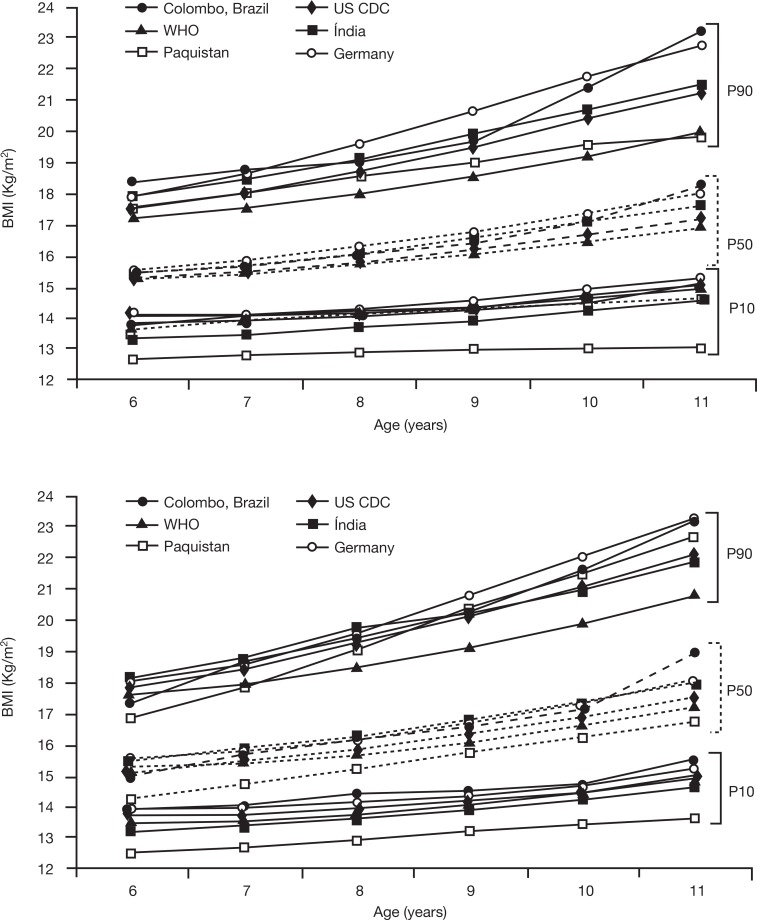
Comparison of the 10th, 50th, and 90th percentile values for body mass index in children from Colombo with children from other countries

BMI 10^th^ and 50^th^ percentile values were similar among children from different studies, both in boys and girls. The largest differences between countries occurred in the 90^th^ percentile values. Children from Colombo (Brazil) had similar BMI percentile values than German children and higher than children from other countries, including those from the WHO study and those from United States, India, and Pakistan. This pattern was similar in boys and girls (Fig. 1).

**Figure 2:**
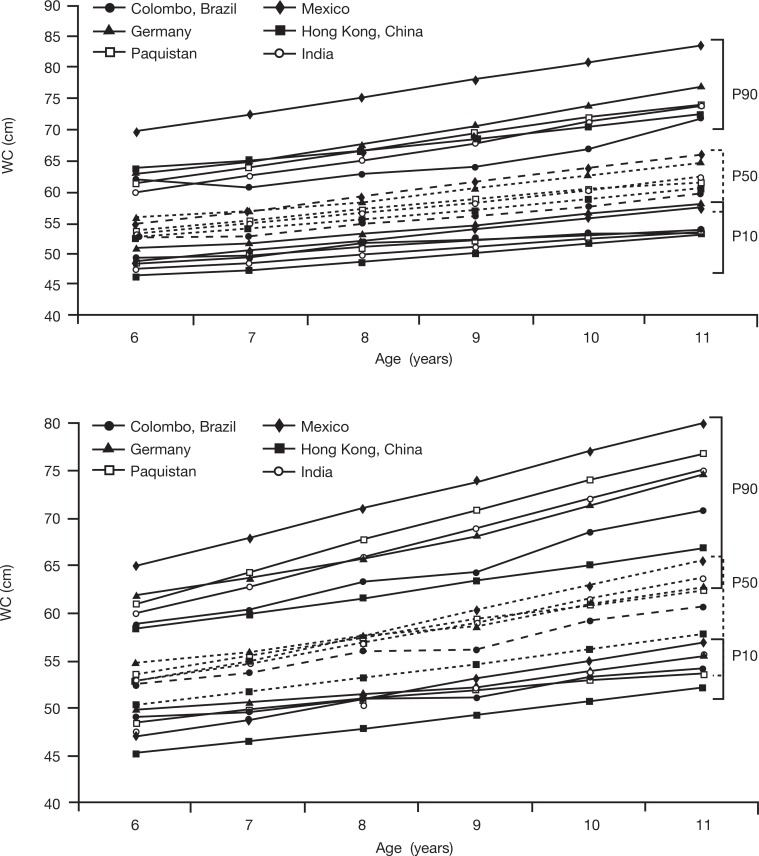
Comparison of the 10th, 50th, and 90th percentile values for waist circumference in children from Colombo with children from other countries

For WC, differences between countries were also more evident in the 90^th^ percentile values. Boys and girls from Colombo (Brazil) had lower age-specific 90^th^ percentile values than children from other countries. Additionally, Mexican boys and girls had the highest 90^th^ percentile values for WC. Boys from Germany and girls from Pakistan also had higher 90^th^ percentile values than children from Colombo (Brazil) and other countries (Fig. 2).

**Figure 3:**
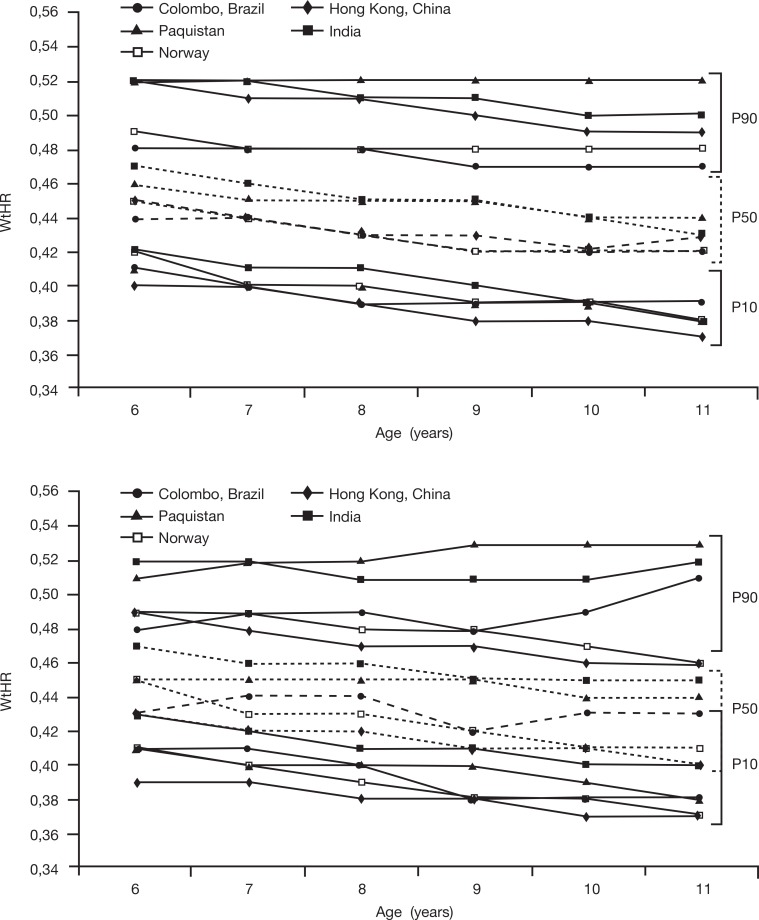
Comparison of the 10th, 50th, and 90th percentile values for waist-to-height ratio in children from Colombo with children from other countries

Differences for WHtR percentile values between children from Colombo (Brazil) and other countries were more evident, considering the 90^th^ percentile values. However, in some cases, the 50^th^ percentile values among children from a country were similar to the 10^th^ percentile values from another countries (e.g., children from Colombo Brazil and Hong Kong had a 50^th^ percentile values ​​very close to the 10^th^ percentile values of children from India). Boys and girls from Colombo (Brazil) had intermediate or lower percentile values in comparison to children from other countries. The highest 90^th^ percentile values for WHtR were observed in Pakistani boys and girls (Fig. 3).

## Discussion

To our knowledge, this was the first study that shows BMI, WC and WHtR percentile values in a sample of Brazilian children and compares them with international data. The comparison of anthropometric indicators of general and abdominal obesity is useful and feasible to identify growth patterns among children from different regions/countries.^18^ Three practical implications can be highlighted with the present study: (1) it allows a better estimate of growth pattern because the LMS method was used, (2) it may show child populations with physical growth trends that are favorable to nutritional disorders (i.e., a child population with elevated anthropometric indices in comparison to others), and (3) it tests the need of anthropometric indices percentile curves for Brazilian children. 

The main evidence of this study was that children from Colombo had higher BMI percentiles values when compared to children from several countries. For example, boys and girls from Colombo had a higher 90^th^ percentile value for BMI than those proposed by CDC^16^ and WHO,^15^ which are frequently used as reference curves. A multifactorial combination (genetic, social, behavioral and economic) may explain the differences between populations in the physical growth pattern.^2^^,^ 27 However, we observed that Brazilian children had higher BMI percentile values than children from countries in which childhood obesity has very large prevalence rates.^16^^,^ 17 These evidences reinforce the importance of public policies to combat and prevent the high BMI among Brazilian children. Interventions in different levels (e.g., improvement of economic condition, nutrition education and physical activity promotion) can contribute to healthy physical growth in Brazilian children and avoid a probable increase of nutritional disorders (e.g., general obesity) in the next years. Considering the WC, children from Colombo had the lower WC percentile values than those from several countries, especially if the 50^th^ and 90^th^ percentile values are observed. These positive findings indicate a WC pattern (especially the 90^th^ percentile value) that is still lower in this sample of Brazilian children than those found in children from countries with concerning abdominal obesity rates.^3^

Often, studies used WC reference curves that were not based on data from their specific children (i.e., studies with Brazilian children used WC reference values from non-Brazilian children).^3^^,^ 28 Because the difference between percentile values, the use of reference curves from other countries (mainly Germany^18^ and Mexico^11^) can underestimate the real proportion of abdominal obesity in children from Colombo (Brazil). Therefore, building WC reference curve with a representative sample of Brazilian children is relevant for monitoring the physical growth among childhood in this country. A reference curve that is based on multinational samples, such as the existing ones for BMI,^15^ will had additional advantages due to the possibility of inter-country comparison. Considering that abdominal obesity has a strong impact on children's health,^4^^,^ 6 ^,^ 9 the development of Brazilian and international reference curves are crucial to design public policies focused on preventing and combating the childhood's abdominal obesity.

WHtR was the only physical growth indicator that was stable or slightly decreased with age among children from Colombo. This trend was also observed in other studies.^10^^,^ 12 ^,^ 14 ^,^ 19 Boys from Colombo had lower percentile values than those noted in most studies.^12^^,^ 14 ^,^ 19 Among girls, percentile values for WHtR were intermediate: lower than the percentile values obtained in some countries^12^^,^ 14 and higher than other ones.^10^^,^ 19 In general, children from Colombo had a healthier WHtR pattern in comparison to children from other countries. In clinical and epidemiological practice, the cutoff point of 0.50 has been frequently used to identify children with high WHtR.^4^^,^ 9 ^,^ 12 ^,^ 14 However, our results and the percentile values observed in other countries (Fig. 3) indicate that this cutoff may not be appropriated to identify abdominal obesity among children, especially in early ages. Therefore, identifying the WHtR cutoffs that may discriminate cardiovascular risk should be stimulated. This is fundamental for monitoring secular trends and the public health impact of abdominal obesity among children in Brazil and other countries.

Some strengths of the present study should be highlighted. Again, we highlighted that this was the first study that includes percentile values for BMI, WC and WHtR in a sample of Brazilian children. Another strength was the use of LMS method for smoothing the percentile values. It allowed to describe the dimensions of the anthropometric indicators and their changes with age, considering statistical parameters that improve the interpretation of physical growth.^24^ Third, all studies were selected because they used the same physical growth indicator, they used the same anatomical point, and they adopted the same statistician procedure (LMS method) for the construction of percentile values.^10^^-^ 20 These similarities were important to able the inter-study comparison. Finally, this study selected children from different regions (urban and rural) and school systems (public and private) from Colombo.

This study also had limitations. The sample of this study included children from a small Brazilian municipality and our results should not be extrapolated to other or all regions of Brazil. Additionally, these percentile values should not be used for nutritional status classification in samples of Brazilian children. Another limitation was the small sample of children at some ages (6 and 11 years of age), which may distort the real percentile values. However, other studies used a similar amount of children at some ages.^10^^,^ 11 ^,^ 14 ^,^ 18 ^,^ 20 Additionally, the trends were consistent between ages, which suggests that the low sample size in some ages did not substantially distort the physical growth pattern and the interpretation of the findings.

In conclusion, clear differences in BMI, WC and WHtR were found among children from Colombo (Brazil) and other countries. Boys and girls from Colombo had higher BMI values than children from other countries, including North American children and the values presented by the WHO. Inversely, children from Colombo (Brazil) had intermediate or lower percentile values ​​for WC and WHtR in comparison to children from other countries. This was a positive result that indicates a growth pattern at lower risk for abdominal obesity.
