# Supplementary material for: Anthropometric indices among schoolchildren from a municipality in Southern Brazil: a descriptive analysis using the LMS method
Source: Rev Paul Pediatr. 2014 Dec;32(4):333–41. doi: 10.1016/j.rpped.2014.04.002 (PMC4311787; doi:10.1016/j.rpped.2014.04.002)
Supplement: Translation

# Translation: Índices antropométricos em escolares de um município no Sul do Brasil: análise descritiva utilizando o método LMS☆

**Authors:** Valter Cordeiro Barbosa, Adair da Silva Lopes, Ricardo Rosa Fagundes, Wagner de Campos

**Affiliations:** a Universidade Federal de Santa Catarina (UFSC), Florianópolis, SC, Brasil; b Universidade Federal do Paraná (UFPR), Curitiba, PR, Brasil

**Keywords:** Desenvolvimento infantil, Antropometria, Estado nutricional, Estudo transversal, Crianças

## Abstract

**OBJETIVO:**

Descrever os valores percentílicos do índice de massa corporal (IMC), circunferência da cintura (CC) e relação cintura-estatura (RCEst) em crianças de Colombo, Brasil, e compará-los com os de crianças de outros países.

**MÉTODOS:**

Estudo transversal com amostra probabilística de 2.035 crianças de 6 a 11 anos de idade. Valores percentílicos do IMC, CC e RCEst, suavizados e específicos para sexo e idade, foram calculados utilizando o método LMS. Os percentis 10, 50 e 90 foram comparados com dados de diferentes países.

**RESULTADOS:**

Houve uma clara tendência de aumento do IMC e da CC com a idade, em ambos os sexos. Os valores da RCEst permaneceram estáveis com o avanço da idade, em meninos e meninas. A comparação do padrão de crescimento mostrou claras diferenças entre os países. Meninos e meninas deste estudo tiveram elevados valores de percentil 90 para o IMC, semelhantes aos de crianças alemãs e superiores aos de crianças americanas e do padrão da Organização Mundial de Saúde. Contudo, as crianças deste estudo tiveram valores intermediários para a CC e RCEst, em comparação com crianças de outros países.

**CONCLUSÃO:**

Valores elevados para IMC e intermediários para CC e RCEst foram observados, em comparação às crianças de outros países. Intervenções em diferentes níveis sociais podem evitar uma provável elevação de disfunções nutricionais (principalmente obesidade geral) nos próximos anos.

## Introdução

O padrão de crescimento corporal indica o desenvolvimento do corpo "aceitável" e "esperado" durante a infância, o qual todas as crianças devem alcançar.^1^ No entanto, a ocorrência de distúrbios nutricionais relacionados ao crescimento da criança, tal como a obesidade geral ou centralizada (abdominal), reflete a interação entre fatores ambientais desfavoráveis ​​e/ou predisposição genética.^2^ Como a obesidade geral e abdominal estão cada vez mais presentes em crianças^3^ e aumentam as chances de complicações metabólicas precoces^4^^,^ 5 e futuras,^6^ o monitoramento do crescimento físico é crucial na promoção da saúde das crianças. 

A combinação de diferentes medidas antropométricas (por exemplo, peso, altura ou circunferência da cintura [CC]) ou indicadores (por exemplo, índice de massa corporal [IMC] e relação cintura-estatura [RCEst]) tem sido frequentemente utilizada em estudos sobre o crescimento e saúde da criança.^6^^-^ 8 Desses indicadores antropométricos, o IMC é o mais frequentemente utilizado para identificar o padrão de crescimento físico e estado nutricional na prática clínica e epidemiológica, pois é um indicador simples e de baixo custo, bem como um forte preditor de saúde da criança.^7^^,^ 8 A CC representa o acúmulo de gordura abdominal e visceral e prevê fatores de risco cardiovascular, assim como ou até mesmo melhor do que o IMC.^4^^,^ 6 Outros estudos enfatizaram que RCEst está fortemente associada a fatores de risco cardiovascular em idades menores.^5^^,^ 9 Assim, o uso desses diferentes métodos antropométricos permite uma melhor estimativa do padrão de crescimento infantil e estado nutricional durante a infância. 

Curvas percentílicas de referência foram desenvolvidas para mostrar o padrão de crescimento e estado nutricional das populações pediátricas de diferentes países.^10^^-^ 20 Estas curvas utilizam o método LMS, um procedimento estatístico para uma estimativa mais robusta de valores percentílicos, especialmente se as variáveis ​​de crescimento físico não têm uma distribuição simétrica na população.^21^^,^ 22

Alguns estudos também utilizaram o método LMS para comparar massa corporal e percentis de altura de crianças brasileiras com as de outros países.^23^^,^ 24 No entanto, uma comparação dos valores de percentis de IMC, CC e RCEst entre as crianças brasileiras e crianças de outros países é desconhecida. Um estudo com esses diferentes indicadores antropométricos pode representar uma melhor estimativa do padrão de crescimento físico entre as crianças brasileiras. Além disso, é necessário testar se há diferença antropométrica entre as crianças do Brasil e aquelas desses países, a fim de identificar padrões distintos de crescimento e testar a necessidade das curvas percentílicas antropométricas para as crianças brasileiras.^18^ Finalmente, essa comparação pode identificar se uma população infantil tem tendências de crescimento físico (para condições ambientais e genéticas) que favorecem distúrbios nutricionais, como a obesidade geral e abdominal.^2^ Essas questões são importantes para o desenvolvimento de políticas públicas destinadas a reduzir distúrbios nutricionais em crianças brasileiras. 

Portanto, o objetivo desse estudo foi determinar o padrão de crescimento físico (IMC, CC e RCEst) em crianças em idade escolar da cidade de Colombo, estado do Paraná, sul do Brasil, e compará-lo com o crescimento físico de crianças de outros países. 

## Método

Esse foi um estudo transversal, realizado na cidade de Colombo, estado do Paraná, sul do Brasil. Esse município está localizado ao norte da região metropolitana de Curitiba (capital do estado) e seu Índice de Desenvolvimento Humano (IDH 2000) é 0,764, o 107º entre os 399 municípios no estado do Paraná. Colombo tinha 27.000 crianças matriculadas em aulas regulares da 1ª a 5ª séries em escolas públicas e privadas. Essa foi a população de estudo. 

Os seguintes parâmetros estatísticos foram considerados para estimar o tamanho da amostra: (1) nível de confiança de 95%, (2) erro de amostragem de 3%, (3) prevalência da obesidade (pelo menos um indicador de obesidade) de 50%, a qual considera a máxima variância e superestima o tamanho da amostra, e (4) efeito do desenho de 1,4.^25^ Assim, o tamanho mínimo da amostra do estudo foi estimado em 1.978 crianças. Uma margem de 20% foi adicionada para possíveis perdas e recusas durante a coleta de dados. Portanto, a amostra estimada incluiu 2.400 crianças. 

As escolas foram agrupadas em três estratos: escolas públicas da área urbana, escolas públicas na área rural, e escolas particulares. Em primeiro lugar, foi realizada uma seleção aleatória de escolas em cada um dos três estratos. O número de escolas por estrato foi calculado considerando-se a proporcionalidade das crianças em cada estrato. Todas as escolas selecionadas foram convidadas e aceitaram participar do estudo. Em segundo lugar, todas as crianças matriculadas em turmas do 1° ao 5° ano do ensino fundamental foram convidadas a participar do estudo. Visitamos 14 escolas, o que incluiu um total de 138 classes e 2.750 crianças convidadas a participar desse estudo. 

As medidas antropométricas (peso, estatura e CC) foram obtidas de março a setembro de 2012. Cada criança foi avaliada individualmente, a fim de minimizar ressalvas. Todas as medições foram realizadas por um único avaliador experiente com o objetivo de excluir erros interexaminador. Dois professores de Educação Física realizaram a anotação de dados antropométricos. 

Os materiais utilizados nesse estudo foram: fita métrica (Easyread Cateb(r), São Paulo, Brasil) (0,1cm de largura) fixa em uma parede que não tinha rodapé para determinar a altura; balança digital (Wiso(r), Santa Catarina, Brasil) (resolução de 100g e capacidade de 150kg) para medir o peso corporal; e uma trena de metal (Cescorf(r), Rio Grande do Sul, Brasil) (resolução de 0,1cm) para avaliar a CC. A altura e a massa corporal foram medidas de acordo com o protocolo padrão.^26^ Para as medições de altura e peso corporal, a criança foi avaliada na posição de pé, sem sapatos e vestindo o uniforme de Educação Física. Medidas de peso e altura foram utilizadas para calcular o IMC (kg/m²). O IMC foi classificado por sexo e idade de acordo com os valores propostos pela Organização Mundial da Saúde (OMS).^15^

A CC foi medida na posição em pé no ponto médio entre a margem costal inferior e a crista ilíaca.^6^ Duas medidas foram obtidas de cada criança e a média delas foi calculada (coeficiente de correlação intraclasse=0,99). A relação cintura-estatura (RCEst=CC/altura) foi calculada.^6^

A idade foi calculada considerando-se a diferença entre o aniversário e a data de coleta. O seguinte critério foi usado para determinar o intervalo entre as idades: 6,0-6,9=6; 7,0-7,9=7 anos; 8,0-8,9=8; 9,0-9,9=9 anos; 10,0-10,9=10 anos, e 11,0-11,9=11 anos. Sexo, tipo de escola, turno, grau e localização da escola foram determinados de acordo com as informações obtidas a partir de cada conselho escolar.

As estatísticas descritivas foram baseadas em média e desvio padrão para as variáveis ​​contínuas, e em frequências absolutas e relativas para as variáveis ​​categóricas. Foram construídas curvas percentílicas específicas de idade e sexo para as variáveis ​​antropométricas (IMC, CC e RCEst), utilizando o método LMS.^21^^,^ 22 O método LMS presume que, para os dados positivos e independentes, a transformação de Box-Cox para cada idade pode ser empregada para normalizar a distribuição dos valores de cada uma das variáveis ​​antropométricas. Todos os valores percentuais específicos de sexo e idade para IMC, CC e RCEst (3, 10, 25, 50, 75, 90 e 97) foram suavizados com o programa *LMSChartmaker Light* versão 2.3 (The Institute of Child Health, Londres: www.healthforallchildren.co.uk). 

Foram construídos gráficos que incluíram os valores percentuais 10, 50 e 90 das crianças desse estudo e de outros estudos. Valores percentuais de IMC foram comparados com dados da OMS,^15^ US Center for Disease Control and Prevention,^16^ Paquistão,^20^ Índia13 e Alemanha.^17^ Os valores percentuais da CC foram comparados com dados da Alemanha,^18^ Paquistão,^14^ México,^11^ Hong Kong^19^ e Índia.^12^ Finalmente, valores percentuais de RCEst foram comparados com valores da Índia,^12^ Hong Kong,^19^ Paquistão^14^ e Noruega.^10^ Esses estudos foram escolhidos considerando: (1) crianças com faixa etária semelhante à desse estudo; (2) mesmos procedimentos estatísticos para a construção das curvas percentílicas (método LMS); (3) presença dos valores percentuais 10, 50 e 90, e (4) mesmos protocolos para medidas antropométricas. 

O Comitê de Ética em Pesquisa da Universidade Federal do Paraná aprovou o estudo (CAAE: 5371.0.000.091-10). Cada escola deu sua permissão formal para coletar os dados. Cada um dos pais/responsáveis de cada criança assinou um termo de consentimento para a participação no estudo. 

## Resultados

Entre as 2.750 crianças convidadas a participar do estudo, 26% não retornaram o formulário de consentimento assinado pelos pais/responsáveis ​​ou se recusaram a participar. Essas crianças não foram avaliadas. Além disso, uma criança com 15 anos de idade foi excluída. Não houve perdas devido ao preenchimento incompleto de dados. Assim, a amostra final foi composta por 2.035 crianças (1.016 meninos e 1.019 meninas) que tinham uma idade média de 8,9 ± 1,4 anos de idade. A maior parte da amostra foi composta por alunos que estudavam no período da manhã (55,4%), em escolas públicas (63,3%) e em escolas localizadas na área urbana. De acordo com os critérios da OMS, 0,8% das crianças apresentavam baixo peso, 19,4% apresentavam sobrepeso e 6,0% eram obesos. 

Considerando-se os valores percentuais suavizados e específicos para sexo e idade, o IMC aumentou com a idade, tanto em meninos quanto em meninas. O IMC mostrou tendência a ser semelhante em meninos e meninas até os 10 anos de idade. Aos 11 anos, as meninas apresentavam valores de IMC mais elevados do que os meninos. A CC teve um ligeiro aumento até os 8 anos de idade. Após essa idade, houve um aumento mais rápido da CC tanto para os meninos quanto para as meninas. A CC foi semelhante em meninos e meninas até os 8 anos de idade. As meninas apresentaram uma CC mais elevada do que os meninos em idades posteriores. A RCEst manteve-se estável até os 9 anos de idade. Foram observadas reduções mínimas nos valores de RCEst em idades posteriores. Essa tendência foi semelhante em meninos e meninas (tabela 1). 

**Tabela 1:** Valores percentuais suavizados específicos de sexo e idade para o índice de massa corporal, circunferência da cintura e relação cintura-estatura

Os percentis 10 e 50 de IMC foram semelhantes entre as crianças de diferentes estudos, tanto em meninos quanto em meninas. As maiores diferenças entre os países ocorreram nos valores do percentil 90. As crianças de Colombo (Brasil) apresentaram percentis de IMC semelhantes aos de crianças alemãs e maiores do que de crianças de outros países, incluindo as crianças do estudo da OMS e dos Estados Unidos, Índia e Paquistão. Este padrão foi semelhante em meninos e meninas (fig. 1).

**Figura 1:** Comparação dos valores de percentis 10, 50, e 90 para índice de massa corporal entre crianças de Colombo e crianças de outros países

Para a CC, as diferenças entre os países também foram mais evidentes nos valores de percentil 90. Os meninos e meninas de Colombo (Brasil) apresentaram valores de percentil 90 mais baixos específicos para idade do que as crianças de outros países. Além disso, os meninos e meninas mexicanas apresentaram os maiores valores de percentil 90 para CC. Meninos da Alemanha e meninas do Paquistão também apresentaram valores de percentil 90 mais elevados do que as crianças de Colombo (Brasil) e de outros países (fig. 2). 

**Figura 2:** Comparação dos valores de percentis 10, 50, e 90 para circunferência da cintura entre crianças de Colombo e crianças de outros países

As diferenças para valores percentuais da RCEst entre crianças de Colombo (Brasil) e de outros países foram mais evidentes, considerando os valores de percentil 90. No entanto, em alguns casos, os valores de percentil 50 entre as crianças de um país foram similares aos valores de percentil 10 de outros países (por exemplo, as crianças de Colombo Brasil e Hong Kong tiveram valores de percentil 50 muito próximos dos valores de percentil 10 de crianças da Índia). Meninos e meninas de Colombo (Brasil) apresentaram valores de percentil intermediários ou mais baixos em comparação com as crianças dos outros países. Os maiores valores de percentil 90 para RCEst foram observados em meninos e meninas paquistanesas (fig. 3). 

**Figura 3:** Comparação dos valores de percentis 10, 50, e 90 para razão cintura-altura entre crianças de Colombo e crianças de outros países.

## Discussão

Em nosso conhecimento, esse foi o primeiro estudo que mostra os valores percentuais do IMC, CC e RCEst em uma amostra de crianças brasileiras e os compara com dados internacionais. A comparação dos indicadores antropométricos de obesidade geral e abdominal é útil e viável para identificar padrões de crescimento entre as crianças de diferentes regiões/ países^18^ Três implicações práticas podem ser destacadas com o presente estudo: (1) ele permite uma melhor estimativa do padrão de crescimento porque o método LMS foi utilizado, (2) pode demonstrar populações infantis com tendências de crescimento físico que são favoráveis ​​a distúrbios nutricionais (ou seja, uma população de crianças com índices antropométricos elevados em comparação com as outras), e (3) ele testa a necessidade das curvas percentílicas dos índices antropométricos para crianças brasileiras. 

A principal evidência do estudo foi que as crianças de Colombo apresentaram maiores valores percentis de IMC quando comparadas a crianças de vários países. Por exemplo, meninos e meninas de Colombo tinham um valor de percentil 90 para o IMC maior do que os propostos pelo CDC^16^ e pela OMS,^15^ que são frequentemente usados ​​como curvas de referência. Uma combinação multifatorial (fatores genéticos, sociais, comportamentais e econômicos) pode explicar as diferenças entre as populações em relação ao padrão de crescimento físico.^2^^,^ 27 No entanto, observamos que as crianças brasileiras apresentaram valores percentuais mais elevados de IMC do que as crianças dos países em que a obesidade infantil tem grandes taxas de prevalência.^16^^,^ 17 Essas evidências reforçam a importância de políticas públicas para combater e prevenir o IMC elevado entre as crianças brasileiras. Intervenções em diferentes níveis (por exemplo, a melhoria da condição econômica, educação nutricional e promoção da atividade física) podem contribuir para o crescimento físico saudável em crianças brasileiras e evitar um provável aumento de distúrbios nutricionais (por exemplo, obesidade geral) nos próximos anos. 

Considerando a CC, as crianças de Colombo tinham os menores valores percentuais para CC do que aquelas provenientes de vários países, especialmente se os valores percentis 50 e 90 forem observados. Esses resultados positivos indicam um padrão de CC (especialmente o valor do percentil 90) que ainda é menor nessa amostra de crianças brasileiras do que os encontrados em crianças de países com taxas alarmantes de obesidade abdominal.3 

Com frequência os estudos utilizaram curvas de referência para CC que não foram baseadas em dados específicos de suas crianças (ou seja, os estudos com crianças brasileiras usaram valores de referência de CC de crianças não brasileiras).^3^^,^ 28 Por conta da diferença entre os valores percentuais, o uso de curvas de referência de outros países (principalmente Alemanha^18^ e México^11^) pode subestimar a proporção real da obesidade abdominal em crianças de Colombo (Brasil). Portanto, a construção da curva de referência da CC com uma amostra representativa de crianças brasileiras é relevante para o monitoramento do crescimento físico da população infantil no país. A curva de referência que é baseada em amostras multinacionais, como as existentes para o IMC,^15^ terá vantagens adicionais graças à possibilidade de comparação entre países. Considerando que a obesidade abdominal tem um forte impacto na saúde das crianças,^4^^,^ 6 ^,^ 9 o desenvolvimento de curvas de referência nacionais e internacionais são cruciais para elaborar políticas públicas voltadas para a prevenção e combate à obesidade abdominal na infância. 

A RCEst foi o único indicador de crescimento físico que ficou estável ou diminuiu ligeiramente com a idade entre as crianças de Colombo. Essa tendência também foi observada em outros estudos.^10^^,^ 12 ^,^ 14 ^,^ 19 Os meninos de Colombo apresentaram valores percentuais mais baixos do que os observados na maioria dos estudos^12^^,^ 14 ^,^ 19 Entre as meninas, os valores percentuais para RCEst foram intermediários: menor que os valores percentuais obtidos em alguns países^12^^,^ 14 e maior do que em outros.^10^^,^ 19 Em geral, as crianças de Colombo tinham um padrão mais saudável de RCEst em comparação com as crianças dos outros países. Na prática clínica e epidemiológica, o ponto de corte de 0,50 tem sido frequentemente usado para identificar as crianças com RCEst alta.^4^^,^ 9 ^,^ 12 ^,^ 14 No entanto, os nossos resultados e os valores percentuais observados em outros países (fig. 3) indicam que esse ponto de corte pode não ser apropriado para identificar obesidade abdominal em crianças, especialmente em idades mais jovens. Portanto, a identificação dos pontos de corte da RCEst que podem discriminar risco cardiovascular deve ser estimulada. Isso é fundamental para monitorar tendências seculares e o impacto na saúde pública da obesidade abdominal em crianças no Brasil e em outros países. 

Alguns pontos fortes do presente estudo devem ser destacados. Mais uma vez, destacamos que esse foi o primeiro estudo a incluir valores de percentil para IMC, CC e RCEst em uma amostra de crianças brasileiras. Outro ponto forte foi a utilização do método LMS para suavizar os valores percentuais. Ele permitiu descrever as dimensões dos indicadores antropométricos e suas alterações com a idade, considerando os parâmetros estatísticos que melhoram a interpretação do crescimento físico.^24^ Em terceiro lugar, todos os estudos foram selecionados porque eles usaram o mesmo indicador de crescimento físico, usaram o mesmo ponto anatômico, e adotaram o mesmo procedimento estatístico (método LMS) para a construção de valores percentuais.^10^^-^ 20 Essas semelhanças foram importantes para permitir a comparação interestudo. Finalmente, esse estudo selecionou crianças de diferentes regiões (urbanas e rurais) e os sistemas de ensino (público e privado) de Colombo. 

Esse estudo também teve limitações. A amostra do estudo incluiu crianças de um pequeno município brasileiro e nossos resultados não devem ser extrapolados para outras ou todas as regiões do Brasil. Além disso, estes valores percentuais não devem ser utilizados para classificar o estado nutricional em amostras de crianças brasileiras. Outra limitação foi a pequena amostra de crianças em algumas idades (6 e 11 anos de idade), o que pode distorcer os valores percentuais reais. No entanto, outros estudos utilizaram um número similar de crianças em algumas idades.^10^^,^ 11 ^,^ 14 ^,^ 18 ^,^ 20 Além disso, as tendências foram consistentes entre as idades, o que sugere que o tamanho reduzido da amostra em algumas idades não distorce substancialmente o padrão de crescimento físico e a interpretação dos resultados. 

Em conclusão, diferenças claras no IMC, CC e RCEst foram encontradas entre as crianças de Colombo (Brasil) e outros países. Meninos e meninas de Colombo tiveram valores de IMC mais elevados do que as crianças de outros países, incluindo crianças norte-americanas e os valores apresentados pela OMS. Inversamente, as crianças de Colombo (Brasil) apresentaram valores percentuais intermediários ou mais baixos para CC e RCEst em comparação com as crianças dos outros países. Esse foi um resultado positivo que indica um padrão de crescimento com menor risco de obesidade abdominal.
